# Loss of STAT1 protects hair cells from ototoxicity through modulation of STAT3, c-Jun, Akt, and autophagy factors

**DOI:** 10.1038/cddis.2015.362

**Published:** 2015-12-17

**Authors:** S Levano, D Bodmer

**Affiliations:** 1 Department of Biomedicine, University Hospital Basel, Basel, Switzerland; 2 Clinic for Otolaryngology, Head and Neck Surgery, University Hospital Basel, Basel, Switzerland

## Abstract

Hair cell damage is a side effect of cisplatin and aminoglycoside use. The inhibition or attenuation of this process is a target of many investigations. There is growing evidence that STAT1 deficiency decreases cisplatin-mediated ototoxicity; however, the role of STAT function and the molecules that act in gentamicin-mediated toxicity have not been fully elucidated. We used mice lacking STAT1 to investigate the effect of STAT1 ablation in cultured organs treated with cisplatin and gentamicin. Here we show that ablation of STAT1 decreased cisplatin toxicity and attenuated gentamicin-mediated hair cell damage. More TUNEL-positive hair cells were observed in explants of wild-type mice than that of STAT1^−/−^ mice. Although cisplatin increased serine phosphorylation of STAT1 in wild-type mice and diminished STAT3 expression in wild-type and STAT1^−/−^ mice, gentamicin increased tyrosine phosphorylation of STAT3 in STAT1^−/−^ mice. The early inflammatory response was manifested in the upregulation of TNF-*α* and IL-6 in cisplatin-treated explants of wild-type and STAT1^−/−^ mice. Expression of the anti-inflammatory cytokine IL-10 was altered in cisplatin-treated explants, upregulated in wild-type explants, and downregulated in STAT1^−/−^ explants. Cisplatin and gentamicin triggered the activation of c-Jun. Activation of Akt was observed in gentamicin-treated explants from STAT1^−/−^ mice. Increased levels of the autophagy proteins Beclin-1 and LC3-II were observed in STAT1^−/−^ explants. These data suggest that STAT1 is a central player in mediating ototoxicity. Gentamicin and cisplatin activate different downstream factors to trigger ototoxicity. Although cisplatin and gentamicin triggered inflammation and activated apoptotic factors, the absence of STAT1 allowed the cells to overcome the effects of these drugs.

The process of auditory sensorineural damage implicates a variety of intracellular events caused by aging, noise exposure, aminoglycoside antibiotics, or the chemotherapeutic agent cisplatin. The mechanisms underlying the ototoxic effects of cisplatin and gentamicin are not yet completely understood. Their ototoxicity likely involves morphological changes and the modulation of pro- and anti-apoptotic cell responses.^1^

Activation of oxidative stress and the inflammatory response are common effects of cisplatin- and gentamicin-induced ototoxicity.^2^ Cisplatin increased the early release of pro-inflammatory cytokines in HEI-OC1 cells and in the cochlea of cisplatin-injected rats.^3^ Similarly, gentamicin induced the production of pro-inflammatory cytokines in the organ of Corti explants *in vitro*.^4^ The JAK/STAT pathway is one of the best-characterized cellular signaling pathways in the immune system. STAT1, a regulator of cell death, has been reported to be involved in cisplatin-mediated hair cell damage.^5, 6^ Knockdown of the STAT1 gene by means of siRNA, administrated by transtympanic injection in rats and transfection of UB/OC1 cells, reduced cisplatin-induced hair cell death *in vivo* and *in vitro*. Moreover, STAT1 siRNA preserved hearing in cisplatin-treated rats.^5^ Furthermore, STAT1 phosphorylation has been observed in utricles exposed to cisplatin *in vitro*.^6^

The inactivation of STAT1 in other tissues has also demonstrated a protective effect, for example, by enhancing autophagy in STAT1-deficient hearts^7^ or accelerated skeletal muscle regeneration.^8^ Recent findings demonstrated that inhibition of the JAK2/STAT3 signaling pathway protects against noise-induced damage to cochlear tissue^9^ and STAT3/SOCS3 signaling regulate hair cell regeneration.^10^ Generally, STAT1 and STAT3 are reciprocally regulated, and disruption of their balance directs cells from survival to apoptotic cell death or from inflammatory to anti-inflammatory responses.^11^ However, there is no information about the role of STAT1 in gentamicin-induced hair cell damage.

In the present study, we investigated the impact of the genetic ablation of STAT1 on hair cell damage induced by cisplatin and gentamicin. We also examined a subset of cell signaling mediators involved in apoptosis and survival. Our data indicate that STAT1 has an important role in cisplatin- and gentamicin-mediated hair cell death. We observed differences in the expression of STAT1 and STAT3 in the organ of Corti (OC) from wild-type (WT) and STAT1^−/−^ mice exposed to cisplatin or gentamicin. An early inflammatory response was observed in the cisplatin-treated explants. Finally, we demonstrated regulatory changes of Akt, c-Jun, and autophagy factors in OC explants exposed either to cisplatin or gentamicin.

## Results

### Sensory hair cells from STAT1^−/−^ mice are resistant to cisplatin treatment

**Figure 1:**
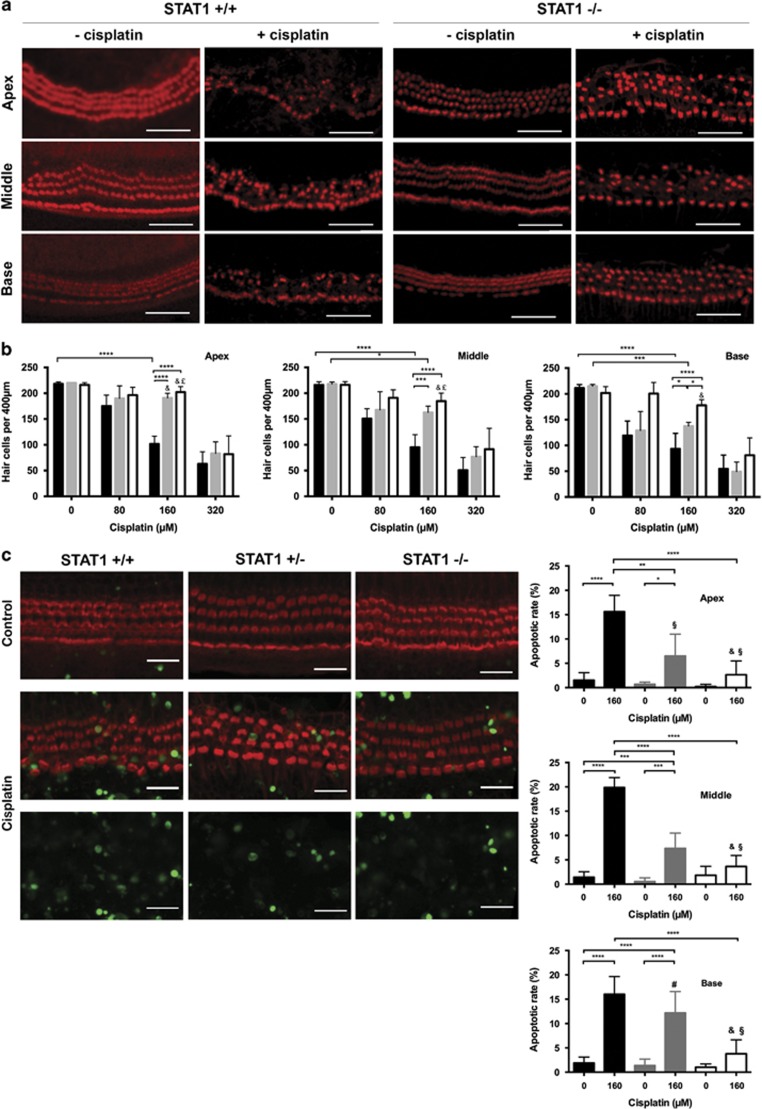
Sensory hair cells from STAT1^−/−^ mice are resistant to cisplatin-mediated ototoxicity. (**a**) Representative confocal images of phalloidin-stained hair cells from all cochlear turns treated with 160 *μ*M cisplatin for 24 h. At this cisplatin concentration, hair cell damage differed significantly between wild-type (STAT1^+/+^) and STAT1^−/−^ mice at all the cochlear turns. Cisplatin induced more hair cell damage in STAT1^+/+^ than in STAT1^−/−^ mice. Scale bar for all figures, 50 *μ*m. (**b**) Quantification of hair cell survival in wild-type (black), heterozygous (gray), and homozygous (white) STAT1 mice. Explants were treated with different concentrations of cisplatin for 24 h. *n*=4–6 explants of each genotype and each condition. (**c**) Representative confocal images of TUNEL and phalloidin double-stained hair cells (middle turn) and quantification of apoptotic rate in wild-type (black), heterozygous (gray), and homozygous (white) STAT1 mice. Explants were treated with 160 *μ*M cisplatin for 24 h. *n*=4–6 explants of each genotype and each condition. Scale bar for all figures, 20 *μ*m. Values shown are mean+S.D. ****P<0.0001, **P<0.01, ***P<0.001, *P<0.05; &, not significant to control of same genotype; §, not significant to non-treated WT sample; #, not significant to cisplatin-treated WT sample; £, not significant to cisplatin-treated heterozygous sample

To investigate the role of STAT1 in hair cell survival, OC explants from STAT1^−/−^, STAT1^+/−^, and WT mice were treated with different cisplatin concentrations for 24 h *in vitro* (Figures 1a and b). Hair cell loss was cisplatin dose dependent. Hair cell survival rates were similar in the basal region of non-treated explants from WT (211±6.58, mean±S.D., *n*=6) and STAT1^−/−^ (202±12.39, *n*=4) mice. At 160 *μ*M cisplatin, hair cell survival in the basal region of WT mice was 93±30.18 (*n*=5); in comparison, hair cell survival was significantly increased in STAT1^−/−^ mice (178±11.03, *n*=6; Figure 1b). A significant increase was also observed in STAT1^+/−^ mice (138±7.23, *n*=4). At this concentration, respective hair cell survival was 95±24.42 and 102±15.18 in the middle and apical regions of WT mice (*n*=5), *versus* 185±15.47 and 202±10.7 in STAT1^−/−^ mice (*n*=6). At the highest concentration used in this study, most of the hair cells were lost and we observed no differences between the mouse groups. The concentration of 160*μ*M cisplatin was used for further experiments.

Hair cell death, apoptosis, was verified by staining DNA fragmentation with TUNEL assay. OC from STAT1^−/−^, STAT1^+/−^, and WT mice were treated with 160 *μ*M cisplatin for 24 h *in vitro*. More TUNEL-positive hair cells were observed in explants of WT mice than that of STAT1^+/−^ and in STAT1^−/−^ mice after cisplatin treatment (Figure 1c).

### Gentamicin-mediated hair cell death was attenuated in STAT1^−/−^ mice

**Figure 2:**
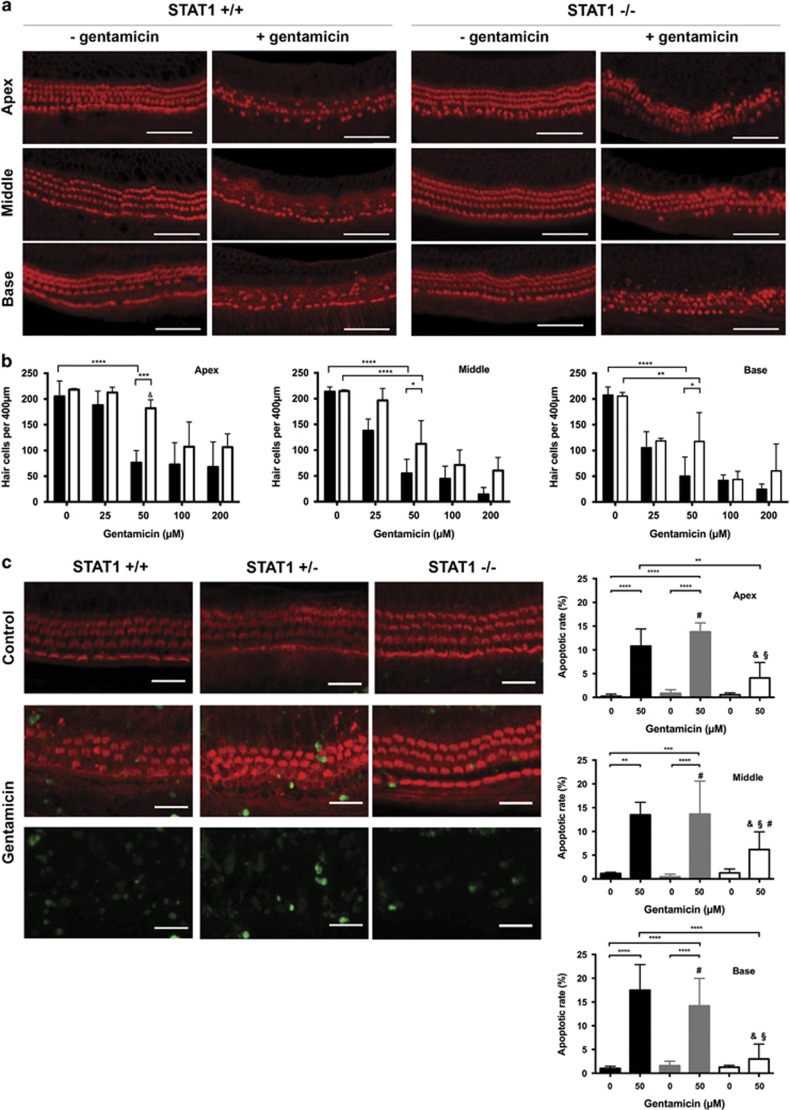
Gentamicin-induced hair cell damage was attenuated in STAT1^−/−^ mice. (**a**) Representative confocal images of phalloidin-stained hair cells from all cochlear turns treated with 50 *μ*M gentamicin for 24 h. At this gentamicin concentration, hair cell damage differed significantly between wild-type and STAT1^−/−^ mice at all the cochlear turns. Gentamicin induced more hair cell damage in STAT1^+/+^ than in STAT1^−/−^ mice. Scale bar for all figures, 50 *μ*m. (**b**) Quantification of hair cell survival in wild-type (black) and homozygous (white) STAT1 mice. Explants were treated with different concentrations of gentamicin for 24 h. *n*=3–5 explants of each genotype and each condition. (**c**) Representative confocal images of TUNEL and phalloidin double-stained hair cells (middle turn) and quantification of apoptotic rate in wild-type (black), heterozygous (gray), and homozygous (white) STAT1 mice. Explants were treated with 50 *μ*M gentamicin for 24 h. *n*=4–6 explants of each genotype and each condition. Scale bar for all figures, 20 *μ*m. Values shown are mean+S.D. ****P<0.0001, ***P<0.001, **P<0.01, *P<0.05; &, not significant to control of same genotype; §, not significant to non-treated WT sample; #, not significant to gentamicin-treated WT sample

To investigate the role of STAT1 in hair cell survival, OC from STAT1^−/−^ and WT mice were treated with different gentamicin concentrations for 24 h *in vitro* (Figures 2a and b). The hair cell survival rates were similar in the basal regions of non-treated explants from WT (208±15.7, mean±S.D., *n*=4) and STAT1^−/−^ (206±7.19, *n*=4) mice. With increasing concentration of gentamicin, hair cell loss was visible in both mice; however, STAT1^−/−^ mice demonstrated greater hair cell survival (Figure 2b). At 25 *μ*M gentamicin, hair cell survival rates were similar in the basal regions of STAT1^−/−^ (105±31.01, *n*=4) and WT mice (119±4.95, *n*=4). At 50 *μ*M gentamicin, hair cell survival dropped to 50±30.07 (*n*=5) in the basal region of WT mice, but was maintained at 118±56.12 (*n*=5) in STAT1^−/−^ mice. Similar hair cell survival rates were observed in the middle (112±45.11) and apical (182±16.37) regions of STAT1^−/−^ mice (*n*=5). The concentration of 50 *μ*M gentamicin was used for further experiments.

Hair cell death was verified by TUNEL assay. OC from STAT1^−/−^, STAT1^+/−^, and WT mice were treated with 50 *μ*M gentamicin for 24 h *in vitro*. More apoptotic hair cells were observed in explants of WT mice than that of STAT1^−/−^ mice after gentamicin treatment (Figure 2c).

### Cisplatin, but not gentamicin, induced STAT1 activation

**Figure 3:**
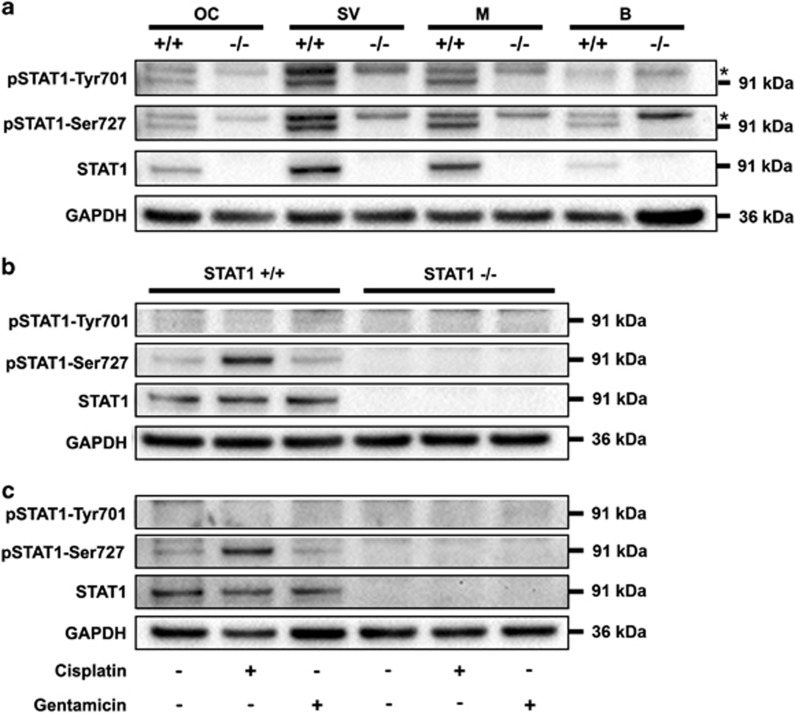
Treatment with cisplatin induced prolonged activation of pSTAT1-Ser727. (**a**) STAT1 expression in the organ of Corti (OC), stria vascularis (SV), modiolus (M), and brain (B) from STAT1 wild-type (+/+) and knockout (−/−) mice. Phosphorylated and non-phosphorylated STAT1 were detected in wild-type tissues. SV exhibited a higher STAT1 content than M and OC. Two western blots were performed, each of them with a pool of six explants for each genotype. Brain was originally used as the control tissue. The asterisks indicate nonspecific bands. (**b**, **c**) Explants were treated with 160 *μ*M cisplatin or 50 *μ*M gentamicin for 6 h (**b**) and 24 h (**c**). Note that pSTAT1-Tyr701 was barely detectable in wild-type tissue. STAT1 expression was induced by cisplatin, but not by gentamicin. GAPDH was used as a loading control. The data are representative of two independent experiments. Six explants were pooled for each genotype and condition

STAT1 and its phosphorylated forms were detected in the inner compartments of cochlea from WT mice, with the stria vascularis demonstrating slightly higher expression than the OC and the modiolus. No protein expression was detected in STAT1^−/−^ cochlea (Figure 3a).

To investigate STAT1 activation, WT and STAT1^−/−^ explants were treated with cisplatin or gentamicin for 6 h (Figure 3b) and 24 h (Figure 3c). No protein expression was detected in STAT1^−/−^ explants (Figures 3b and c). Neither cisplatin nor gentamicin induced tyrosine phosphorylation in cultured organs from WT mice. Cisplatin triggered stronger serine phosphorylation in WT organs, which was prolonged at least for 24 h. In contrast, serine phosphorylation remained unchanged in gentamicin-treated organs.

### Cisplatin, but not gentamicin, reduced STAT3 expression

**Figure 4:**
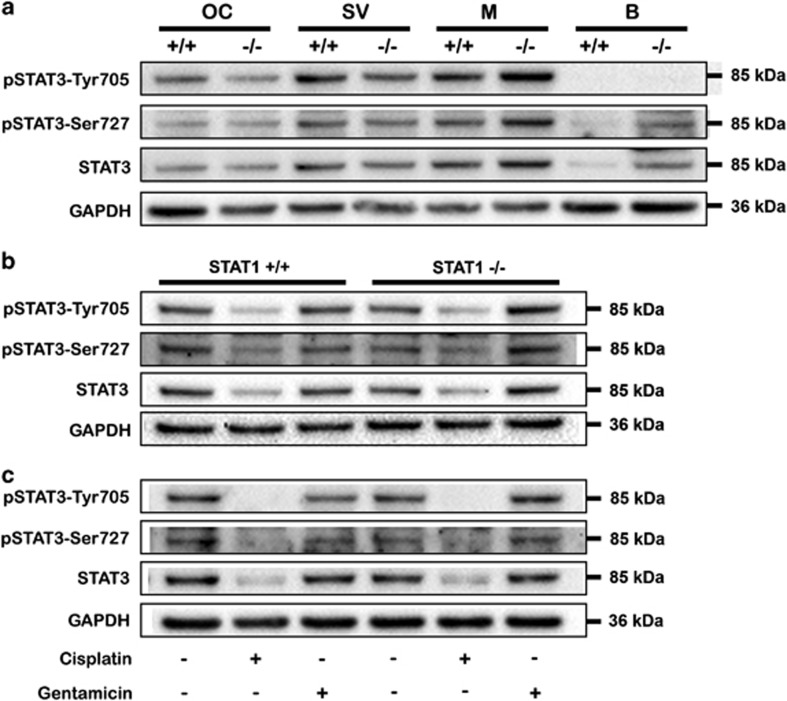
Cisplatin decreased STAT3 expression, and gentamicin induced activation of STAT3. (**a**) STAT3 expression in the organ of Corti (OC), stria vascularis (SV), modiolus (M), and brain (B) from STAT1 wild-type (+/+) and knockout (−/−) mice. No change of STAT3 expression was observed in OC, SV, M, or B from STAT1 wild-type (+/+) or knockout (−/−) mice. Modioli from STAT1^−/−^ mice exhibited higher STAT3 content. Two western blots were performed, each of them with a pool of six explants for each genotype. Brain was originally used as the control tissue. (**b, c**) Explants were treated with 160 *μ*M cisplatin or 50 *μ*M gentamicin for 6 h (**b**) and 24 h (**c**). STAT3 expression and phosphorylation was inhibited by cisplatin, but the expression of pSTAT3-Tyr705 was induced by gentamicin. GAPDH was used as a loading control. The data are representative of two independent experiments. Six explants were pooled for each genotype and condition

The expression of STAT3 and its phosphorylated form was similar in WT and STAT1^−/−^ mice and across the inner compartments, except for the slightly high expression observed in modioli from STAT1^−/−^ mice (Figure 4a).

To investigate STAT3 activation, WT and STAT1^−/−^ explants were treated with cisplatin or gentamicin for 6 h (Figure 4b) and 24 h (Figure 4c). Cisplatin diminished the expression and phosphorylation of STAT3 protein at 6 h and abrogated them for at least 24 h. In contrast, gentamicin did not activate or change STAT3 expression in WT or STAT1^−/−^ organs, except the tyrosine phosphorylation of STAT3 in STAT1^−/−^ explants at 24 h.

### Cisplatin initiates inflammation: upregulation of TNF-*α* and IL-6

**Figure 5:**
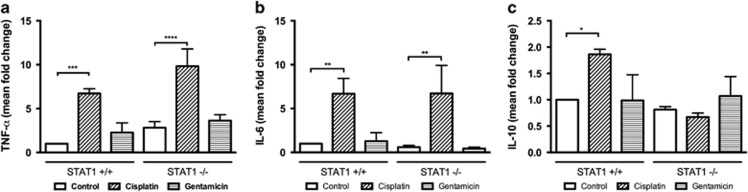
Cytokine expression was regulated by cisplatin. Levels of TNF-*α* (**a**), IL-6 (**b**), and IL-10 (**c**) gene expression in explants from STAT1^+/+^ and STAT1^−/−^ mice. Organs of Corti were exposed to 160 *μ*M cisplatin or 50 *μ*M gentamicin (GM) for 6 h. mRNA expression in the organ of Corti was quantified using real-time PCR and the comparative ΔΔCT method. The values were normalized against GAPDH. Mean fold changes are represented as mean+S.D. relative to untreated (control) explants. Data are from three independent experiments. RT-PCR reactions were performed in triplicate. Five to six explants were pooled for each genotype and per condition. Values shown are mean+S.D. ****P<0.0001, ***P<0.001, **P<0.01, *P<0.05

Because cisplatin and gentamicin have been associated with inflammation, we investigated the expression of pro-inflammatory cytokines in WT and STAT1^−/−^ explants treated with cisplatin and gentamicin at 6 h, a time point at which cell death may not occur. The basal expression of TNF-*α* was 2.8-fold higher in STAT1^−/−^ than in WT mice, however, this relationship did not reach significance (Figure 5a). Cisplatin upregulated the early expression of TNF-*α* by 6.7-fold in WT mice (*P*<0.001) and by 9.8-fold in STAT1^−/−^ mice (*P*<0.0001). Gentamicin increased the early expression of this cytokine by 2.3-fold in WT mice and by 3.6-fold in STAT1^−/−^ mice; however, this difference did not reach statistical significance.

Cisplatin induced early expression of IL-6 in WT and STAT1^−/−^ explants by 6.7-fold (*P*<0.01; Figure 5b). Gentamicin increased IL-6 expression by 1.3-fold in WT and reduced by 0.44-fold in STAT1^−/−^ mice. However, these differences failed to reach statistical significance.

### Cisplatin, but not gentamicin, affected the expression of IL-10

To investigate early expression of the anti-inflammatory cytokine IL-10, we treated WT and STAT1^−/−^ explants with cisplatin or gentamicin for 6 h. Basal IL-10 expression was similar in STAT1^−/−^ mice and in WT mice (Figure 5c). Cisplatin increased IL-10 expression by 1.9-fold in WT mice (*P*<0.05), and reduced it by 0.67-fold in STAT1^−/−^ mice (*P*=0.82). Gentamicin treatment did not change IL-10 expression in WT or STAT1^−/−^ mice.

### Gentamicin activates Akt in STAT1^−/−^ mice

**Figure 6:**
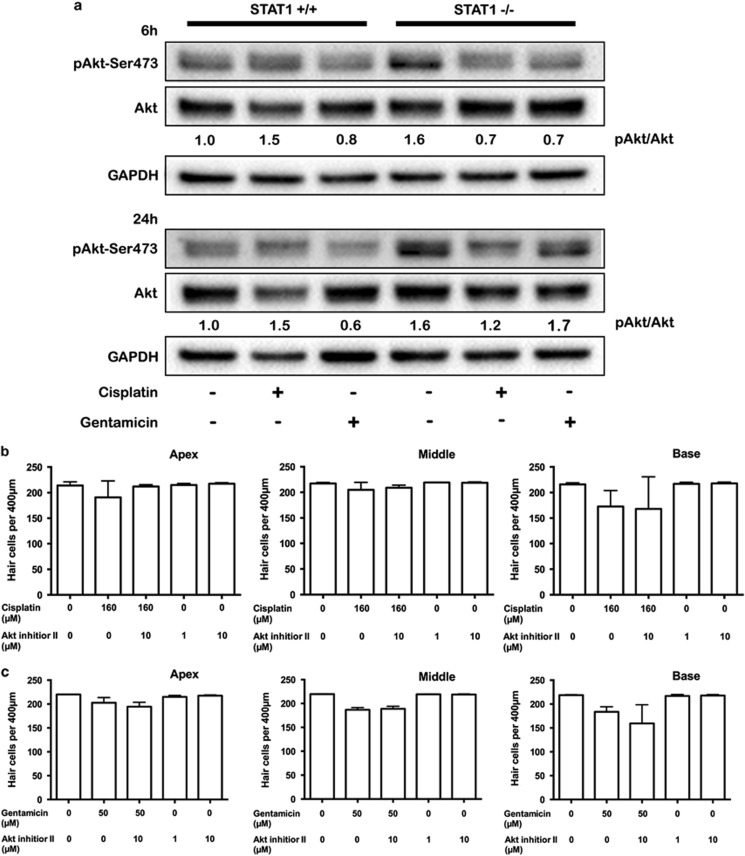
Activation of Akt by gentamicin at 24 h. Explants from STAT1^+/+^ and STAT1^−/−^ mice were treated with 160 *μ*M cisplatin or 50 *μ*M gentamicin for 6 h and 24 h (**a**) and subjected to western blotting. Gentamicin induced Akt expression in STAT1^−/−^ explants. The data are representative of two independent experiments. Six explants were pooled for each genotype and condition. Densitometric analysis of each protein normalized against WT control (untreated) is shown. GAPDH was used as a loading control. (**b**, **c**) Quantification of hair cell survival in explants of STAT1^−/−^ mice. Explants were treated with 160 *μ*M cisplatin (**b**) or 50 *μ*M gentamicin (**c**) in the presence of Akt inhibitor II for 24 h. *n*=4–5 explants of each genotype and each condition. Values shown are mean+S.D

Because phosphorylated Akt participates in hair cell survival pathway, we investigated the activation of Akt in WT and STAT1^−/−^ explants treated with cisplatin or gentamicin for 6 h and 24 h. Akt activation was higher in untreated STAT1^−/−^ explants than in WT mice at 6 h and 24 h (Figure 6a). At 6 h, Akt activation was diminished in cisplatin-treated explants from STAT1^−/−^ mice and in gentamicin-treated explants from WT and STAT1^−/−^ mice. At 24 h, gentamicin induced Akt activation in gentamicin-treated explants from STAT1^−/−^ mice.

To further investigate whether suppression of phosphorylated Akt affects the level of cell survival in cisplatin- and gentamicin-treated STAT1^−/−^ explants, the Akt inhibitor II was used in combination with cisplatin or gentamicin. Akt inhibitor II alone (1 and 10 *μ*M) or in combination with gentamicin or cisplatin did not affect hair cell survival in explants of STAT1^−/−^ mice (Figures 6b and c). Hair cells of STAT1^−/−^ basal turn were more susceptible to gentamicin and Akt inhibitor II treatment than gentamicin alone; however, this did not reach statistical significance (*P*=0.83; Figure 6c).

### Cisplatin and gentamicin activate c-Jun

**Figure 7:**
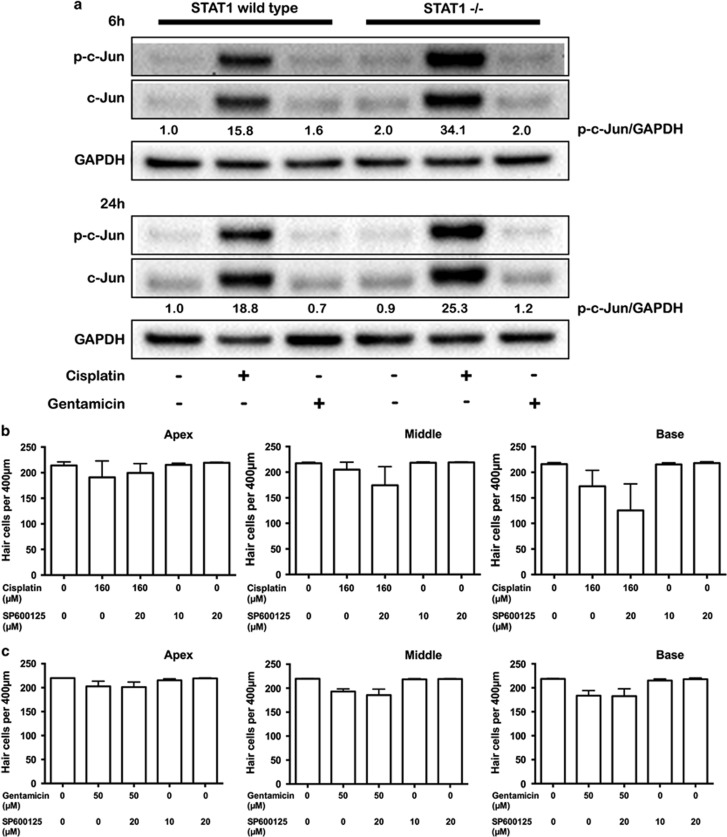
Prolonged activation of c-Jun by cisplatin. Explants from STAT1^+/+^ and STAT1^−/−^ mice were treated with 160 *μ*M cisplatin or 50 *μ*M gentamicin for 6 h and 24 h (**a**) and subjected to western blotting. Cisplatin induced the sustained expression of c-Jun for at least 24 h. Gentamicin induced early activation of c-Jun, but less pronounced than cisplatin treatment. Because the expression of total c-Jun protein changes, p-c-Jun was normalized against GAPDH, which was used as a loading control. Densitometric analysis of each protein normalized against WT control (untreated) is shown. The data are representative of two independent experiments. Six explants were pooled for each genotype and condition. (**b, c**) Quantification of hair cell survival in explants of STAT1^−/−^ mice. Explants were treated with 160 *μ*M cisplatin (**b**) or 50 *μ*M gentamicin (**c**) in the presence of SP600125 for 24 h. *n*=4–5 explants of each genotype and each condition. Values shown are mean+S.D.

Phosphorylation of c-Jun has been observed in cisplatin-mediated hair cell death; therefore, we investigated the activation of c-Jun in WT and STAT1^−/−^ mice. The phosphorylation of c-Jun was barely detected in untreated WT explants (Figure 7a). Cisplatin induced strong and sustained activation of c-Jun in WT explants, and even more in explants from STAT1^−/−^ mice, while gentamicin induced transient phosphorylation of c-Jun at a lower level than cisplatin activation.

To further investigate whether suppression of phosphorylated c-Jun affects the level of cell survival in cisplatin- and gentamicin-treated STAT1^−/−^ explants; SP600125, a c-Jun N-terminal kinase (JNK) inhibitor, was used in combination with these drugs. SP600125 alone (10 and 20 *μ*M) or in combination with gentamicin or cisplatin did not affect hair cell survival in the explants of STAT1^−/−^ mice (Figures 7b and c). Hair cells of STAT1^−/−^ basal turn were more susceptible to cisplatin and SP600125 treatment than cisplatin alone; however, this did not reach statistical significance (*P*=0.60; Figure 7b).

### Autophagy is triggered by cisplatin and gentamicin

**Figure 8:**
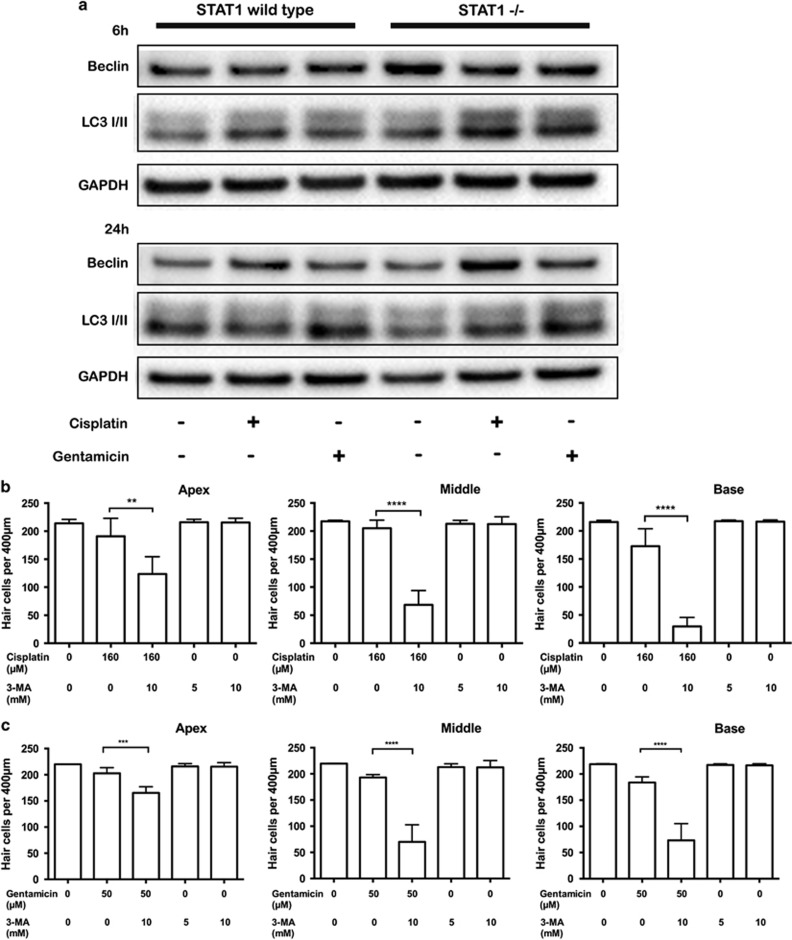
Cisplatin and gentamicin induce autophagy in STAT1^−/−^ mice. Explants from STAT1^+/+^ and STAT1^−/−^ mice were treated with 160 *μ*M cisplatin or 50 *μ*M gentamicin for 6 h and 24 h (**a**). After treatment with cisplatin or gentamicin, Beclin-1 expression was higher in STAT1^−/−^ than in wild-type explants. A difference in LC3-II expression between wild-type and STAT1^−/−^ mice was observed at 6 h. The data are representative of two independent experiments. Six explants were pooled for each genotype and condition. GAPDH was used as a loading control. (**b, c**) Quantification of hair cell survival in explants of STAT1^−/−^ mice. Explants were treated with 160 *μ*M cisplatin (**b**) or 50 *μ*M gentamicin (**c**) in the presence of 3-MA for 24 h. 3-MA in combination with cisplatin or gentamicin induced more hair cell damage. *n*=4–5 explants of each genotype and each condition. Values shown are mean+S.D. ****P<0.0001, ***P<0.001, **P<0.01

Because STAT1 and STAT3 are associated with autophagy, we analyzed autophagy factors that might modulate hair cells' escape from cell death in response to ototoxic drugs. The conversion of LC3-II was higher in the treated explants from STAT1^−/−^ mice than in those from WT mice at 6 h (Figure 8a). At 24 h, high Beclin-1 expression was maintained in cisplatin and gentamicin-treated explants from STAT1^−/−^. LC3-II expression did not differ between the two types of mice at 24 h.

To establish the role of Beclin-1 and LC3-II in hair cell survival in STAT1^−/−^ mice, we analyzed the effect of 3-MA, an inhibitor of phosphatidylinositol 3-kinase (PI3K) and widely used as an autophagy inhibitor. Inhibition of autophagy by 3-MA reduced the expression of Beclin-1 and the LC3-II/LC3-I conversion.^12, 13^ Treatment with 3-MA in combination with cisplatin (Figure 8b) or gentamicin (Figure 8c) significantly decreased the number of surviving hair cells in explants of STAT1^−/−^ mice.

## Discussion

Cisplatin and gentamicin are widely used in the clinical setting. Cisplatin is used to treat many types of cancer, whereas gentamicin is used in the treatment of severe and life-threatening infections. Unfortunately, ototoxicity is a serious side effect among patients treated with these drugs. Reducing this toxic effect without interfering with the treatment efficacy of cisplatin and gentamicin is one of the main goals in otoprotective research.

Interest in STAT proteins has increased recently because of their association with protection against ototoxicity. One of the earliest reports examined the effect of cisplatin on utricles of STAT1-deficient mice.^6^ Their and our data found that the degree of hair cell death was cisplatin concentration dependent. Gentamicin-induced hair cell death was also concentration dependent. In agreement with the observed increase in hair cell survival in cisplatin- and gentamicin-treated explants of STAT1^−/−^ mice, less TUNEL-positive cells was detected in treated explants from STAT1^−/−^ mice than that of WT mice. This finding is consistent with previous reports showing that transfected cells or cochlea of injected mice with STAT1 siRNA exposed to cisplatin resulted in reduced hair cell death.^5, 6^ In addition and in contrast to the current concept that gentamicin and cisplatin preferentially affect basal turn over the apical and middle turns, no significant difference in hair cell loss has been observed in our study between the turns of STAT1^−/−^ explants treated with cisplatin and gentamicin, except for the lowest concentration used for cisplatin and gentamicin. Lack of a preference for hair cell loss located at the basal turn after cisplatin treatment has been recently reported in Bmi1^−/−^ mice.^14^ The concentrations of cisplatin (160 *μ*M) and gentamicin (50 *μ*M) were selected to best determine the changes in gene expression between STAT1^−/−^ and WT mice.

Cisplatin-mediated STAT1 phosphorylation has been reported as an early transient event in mouse utricles.^6^ However, prolonged STAT1 activation has been observed in cochlear sections from mice treated by intraperitoneal administration of cisplatin.^5^ In agreement with this last observation, we found prolonged serine phosphorylation of STAT1 protein in explants treated with cisplatin. No changes in STAT1 levels were observed in gentamicin-treated explants, suggesting that STAT1 is not a direct target of gentamicin-mediated ototoxicity.

It has been suggested that STAT1 and STAT3 often have opposite roles with respect to target cells: while STAT1 is considered an apoptotic factor, STAT3 is required for survival.^11^ In our experiments, cisplatin diminished phosphorylation and expression of STAT3 irrespective of STAT1 expression. This observation of STAT3 inhibition is in good agreement with a recent report describing the modulation of Lmo4 by cisplatin in the inner ear. They found that cisplatin decreased Lmo4 and its downstream target, STAT3.^15^ On the other side, in our study, gentamicin-treated OC explants presented transient tyrosine phosphorylation of STAT3 in STAT1^−/−^ mice. Usually, following tyrosine phosphorylation, STAT3 proteins dimerize, translocate into the nucleus, and activate transcription of specific target genes, for example, Bcl2 family members. In the auditory system, overexpression of Bcl2 attenuated gentamicin-induced hair cell death in zebrafish.^16^ In addition, STAT3 interacts with other survival pathways, MEK/MAPK and PI3K/Akt, and in particular a study found that STAT3 and PI3K/Akt pathways mediated survival response of sensory neurons to cytokine.^17^ In fact, cisplatin and gentamicin exert different actions on STAT3 expression and precise regulation of STAT3 in terms of time course seems to be important for its beneficial effects.

The production of pro-inflammatory cytokines is reportedly upregulated after cisplatin treatment.^3, 18^ These cytokines are known to participate in the STAT1 cascade. We found that cisplatin induced the upregulation of TNF-*α* and IL-6 expression in both WT and STAT1^−/−^ mice; moreover, cisplatin increased IL-10 expression in explants of WT mice. The fact that cisplatin activated an early immediate pro-inflammatory and anti-inflammatory cytokine release in WT explants, while an early anti-inflammatory cytokine release was not observed in STAT1^−/−^ mice, suggests that distinct sets of cytokines against ototoxicity are initially activated in WT and STAT1^−/−^ mice. It is known that cytokines activate downstream factors that could exert opposing actions. Indeed, NF-*κ*B pathway, inhibited by IL-10 and activated by IL-6, could function as pro- or anti-apoptotic factor.^1, 19, 20^ In contrast to our finding, previous reports indicate that TNF-*α* is downregulated after the siRNA suppression of STAT1.^5^ Moreover, attenuation of inflammatory cytokine through flurazine protected mouse cochlea against cisplatin toxicity.^21^ However, protection against ototoxicity was not always accompanied by the attenuation of pro-inflammatory cytokines.^22^ These discrepancies are probably related to the fact that most of these studies about cytokines focused on the later stage of hair cell damage. On the other hand, although gentamicin affected the expression of TNF-*α* and IL-6 in STAT1^−/−^ explants, this failed to reach significance. Our observation contrasts with previous report describing the upregulation of pro-inflammatory cytokines by gentamicin in OC explants.^4^ One possible reason for this difference may be the time point used in our experiments and testing various time points may help clarify this topic.

Our data indicated that cisplatin did not promote phosphorylation of Akt. This finding contrasts with two recent studies in HEI-OC1 auditory hair cell line. Kim *et al.*^23^ demonstrated that 20 *μ*M cisplatin induced Akt phosphorylation at 3 h, while Ma *et al.*^24^ reported reduction of Akt phosphorylation after incubation with 100 *μ*M cisplatin for 24 h. The reason for these differences of Akt activation remains unclear; however, it seems that the phosphorylation of Akt is time dependent and not a prolonged action. On the other hand, gentamicin triggered the phosphorylation of Akt in STAT1^−/−^ explants at 24 h in our study. Akt participation in hair cell survival has been reported recently.^25^ Similar results of Akt phosphorylation induced by gentamicin in STAT1^−/−^ explants have been observed in the neurons of STAT1^−/−^ mice after 2 h ischemia/2 h reperfusion.^26^ Moreover, Chung *et al.*^27^ observed the activation of Akt at 6 h after gentamicin treatment and less hair cell loss by combining Akt inhibitor (SH-6) and gentamicin. They suggested that Akt participates in the mechanism underlying hair cell survival. Contrary to our expectations, Akt inhibitor II (SH-5) did not affect the hair cell survival in gentamicin-treated explants of STAT1^−/−^ mice. Although the reason for this discrepancy is unclear, there may be several possibilities, for example the inhibitors did not fully inactivate the proteins in the explants of STAT1^−/−^ mice. Another possibility is that Akt does not need to be phosphorylated to participate in hair cell survival process. Indeed, PI3K/Akt participates in the regulation of autophagy, and Akt interaction is not phosphorylation dependent.^28^

Our data indicated that cisplatin induced an upregulation of c-Jun phosphorylation that was higher in STAT1^−/−^ than in WT. The phosphorylation of c-Jun in STAT1^−/−^ explants was not expected owing to the high percentage of cell survival in our explants suggesting that c-Jun might not exert apoptotic effects in the absence of STAT1, but be involved in hair cell survival. However, the indirect inhibition of c-Jun by using SP600125, inhibitor of JNK, did not affect the hair cell survival in STAT1^−/−^ explants. Similar observation has been described in two studies that demonstrated that although JNK is activated by cisplatin, its inactivation by inhibitors did not protect against cisplatin ototoxicity.^29, 30^ Wang *et al.*^29^ suggested that c-Jun does not participate in the apoptotic pathway of hair cell damage, but does participate in the DNA repair and maintenance of damaged hair cells. Moreover, phosphorylation of c-Jun can also be induced in a JNK-independent manner.^31^ With regard to participation of c-Jun in cell survival, previous *in vitro* study with fibroblast has shown that c-Jun phosphorylation promotes cellular survival by suppressing the expression of PTEN tumor suppressor gene and activation of Akt pathway.^32^ Moreover c-Jun mediates opposing physiological processes depending on its abundance, its dimerization partners, and its interaction with other cofactors.^33^

The transcription factor c-Jun is also reportedly involved in the pathogenesis of gentamicin ototoxicity, owing to the increase of the p-c-Jun/c-Jun ratio^34^ and the protective effect after inhibition of JNK.^35^ Gentamicin slightly induced phosphorylation of c-Jun at 6 h in explants from both WT and STAT1^−/−^ mice. This result is consistent with our previous observation of transient c-Jun activation by gentamicin.^34^ The fact that the transient activation of c-Jun was also detected in STAT1^−/−^ explants may suggest that this activation is a STAT1-independent process and is a response to insult. The survival-promoting effect of c-Jun was tested by using SP600125; however, SP600125 treatment did not affect the hair cell survival in STAT1^−/−^ explants. There are several possibilities that can explain the discrepancy, for example the inhibitors did not fully inactivate the proteins in explants of STAT1^−/−^ mice and/or the phosphorylation of c-Jun was induced in a JNK-independent manner.

Little is known about autophagy in sensory hair cells. In the avian inner ear, autophagy appears to be an active process during otic development.^12^ Furthermore, rapamycin, inducer of autophagy, increased the expression of LC3-II and Beclin-1 and alleviated cisplatin-induced ototoxicity *in vivo*.^36^ In addition, autophagy acts as a protective mechanism against myocardial infarction in STAT1-deficient hearts.^7^ Although the experimental conditions differed between these reports and our study, our data are in agreement with these studies regarding the activation of autophagy proteins owing to ototoxicity. We observed that 3-MA, a widely used autophagy inhibitor, increased cisplatin- and gentamicin-induced hair cell death in explants of STAT1^−/−^ mice. Similarly, previous study has demonstrated that 3-MA blocked the conversion of LC3-I to LC3-II expression and increased the number of TUNEL-positive cells in chicken otic vesicles.^12^ Moreover, 3-MA inhibited lysosomal accumulation of the Akt-Phafin2 complex, a critical step in the induction of autophagy.^28^ These results suggest that cisplatin and gentamicin activate different factors in sensory hair cells and autophagy seems to be an important contributor to prevent cisplatin- and gentamicin-induced ototoxicity.

Although more research is needed to clarify the divergent pathway of ototoxicity and its protection against cisplatin and gentamicin, our report points the mechanisms that should be investigated to further understand the mechanism of ototoxicity of these drugs.

In conclusion, we found that auditory hair cells from STAT1^−/−^ mice are more resistant to cisplatin and gentamicin treatment compared with control animals. Cisplatin upregulated TNF-*α* and IL-6. Moreover, cisplatin induced STAT1 activation and diminished expression and phosphorylation of STAT3. Gentamicin induced transient STAT3 tyrosine phosphorylation and Akt serine phosphorylation in STAT1^−/−^ explants. Finally, cisplatin and gentamicin activated c-Jun and triggered autophagy.

## Materials and Methods

### Drugs and antibodies

Cisplatin, gentamicin, penicillin SP600125, and 3-methyladenine (3-MA) were purchased from Sigma Aldrich Chemie GmbH (Steinheim, Germany). Akt inhibitor II was purchased from Calbiochem (EMD Millipore, Darmstadt, Germany). Primary antibodies were used in the following dilutions: STAT1 at 1/1000, both pSTAT1-Ser727 and -Tyr701 at 1/500, both pSTAT3-Ser727 and -Tyr705 at 1/500, Akt at 1/1000, pAkt-Ser473 at 1/1000, p-c-Jun (Ser73) at 1/500, c-Jun at 1/1000, LC3 at 1/1000 (all from Cell Signaling, Bioconcept, Allschwil, Switzerland), STAT3 at 1/1000 (Santa Cruz Biotechnology, Labforce AG, Nunningen, Switzerland), Beclin-1 at 1/1000 (Novus Biologicals, Littleton, MA, USA), and GAPDH at 1/5000 (Abcam, Labforce AG, Nunningen, Switzerland).

### Mice and genotyping

STAT1-deficient mice (mixed C57BL/6-129/SvEv) lack the DNA binding domain of STAT1, and initial characterization has been described elsewhere.^37^ For all the experiments, the mice used were 4–5 days old. The animals were housed in pathogen-free conditions at the animal facility of the Department of Biomedicine of the University Hospital Basel. All animal experiments were conducted with the approval of Animal Care Committee of the Canton Basel City, Switzerland.

To generate a sufficient quantity of WT and STAT1^−/−^ mice, we separated WT and homozygous siblings from the breeding pair of STAT1^+/−^ mice and subsequently mated WT pairs (STAT1^+/+^ × STAT1^+/+^) and homozygous pairs (STAT1^−/−^ × STAT1^−/−^). PCR amplification of genomic DNA was used to identify mouse genotypes.

### Preparation of organs for culture

Mice were decapitated and the cochlea was dissected from the skull in cold 1 × PBS. The OC explants were placed in Dulbecco's modified Eagle's medium supplemented with 10% fetal bovine serum, 25 mM HEPES, and 30 U/ml penicillin. Explants were incubated at 37 °C and 5% CO_2_. After overnight recovery, explants were exposed to cisplatin or gentamicin for 6 h (RNA and protein expression) and 24 h (hair cell damage and protein expression).

### Hair cell counting

After treatment for 24 h with different concentrations of cisplatin and gentamicin, OC explants were fixed and stained with AlexaFluor 568 phalloidin (Invitrogen AG, Basel, Switzerland). Using a Nikon A1R laser confocal microscope (Nikon AG Instruments, Egg, Switzerland) with a × 20 lens, inner and outer hair cells were counted over a 400-*μ*m distance in the basal, middle, and apical turns of each cochlea (fields were selected randomly). Cells were considered missing if there was a gap in the normal ordered array of hair cells.

Detection of apoptotic hair cells was performed using DeadEnd fluorometric TUNEL assay according to the manufacturer's instructions (Promega, Madison, WI, USA). Apoptotic rate was determined by counting TUNEL-positive cells divided by total cells over a 400- *μ*m distance.

### RNA analysis

For quantitative real-time PCR, OC explants were cultured in the absence or presence of 160 *μ*M cisplatin or 50 *μ*M gentamicin for 6 h. Five to six OC explants were used for each condition and each genotype. After treatment, the five to six OC explants were pooled together as one biological sample and stored in RNA stabilization reagent (QIAGEN, Hombrechtikon, Switzerland) at −20 °C until further use. Total RNA was extracted using an RNeasy Plus Mini kit according to the manufacturer's instructions (QIAGEN). RNA concentration and purity were determined using the Nanodrop ND-1000 (Thermo Fisher Scientific, Reinach, Switzerland). cDNA synthesis was completed using a high-capacity cDNA reverse transcription kit (Applied Biosystems, Foster City, CA, USA). Real-time PCR was performed using a Fast SYBR Master mix kit (Applied Biosystems) and ABI 7500 Fast Real-Time PCR instrument (Applied Biosystems). The following sequence-specific primers were used: TNF-*α* forward 5′-CCACCACGCTCTTCTGTCTAC-3′, TNF-*α* reverse 5′- AGGGTCTGGGCCATAGAACT-3′ (103 base pairs, NM_013693); IL-6 forward 5′-TGATGCACTTGCAGAAAACA-3′, IL-6 reverse 5′-ACCAGAGGAAATTTTCAATAGGC-3′ (109 base pairs, NM_031168); IL-10 forward 5′-ATCGATTTCTCCCCTGTGAA-3′, IL-10 reverse 5′-TGTCAAATTCATTCATGGCCT-3′ (108 base pairs, NM_010548); GAPDH forward 5′-CGTCCCGTAGACAAAATGGT-3′, GAPDH reverse 5′-TTGATGGCAACAATCTCCAC-3′ (110 base pairs, NM_001001303). Melting curves were calculated to ensure primer specificity. Each reaction was performed in triplicate. Relative quantification of mRNA levels was assessed using the 2-ΔΔCT method and normalized against the housekeeping gene GAPDH, as well as against the expression levels of control OC explants from WT mice.

### Immunoblotting

For the preparation of protein lysate from fresh tissues, six microdissected organs of the cochlea (spiral ganglion, OC, and modiolus) were pooled as one biological sample. Mouse brain was collected. The tissues were immediately transferred into T-PER lysis buffer (Thermo Fisher Scientific) containing protease and phosphatase inhibitors.

For the preparation of cultured OC explants, five to six explants were used for each condition and each genotype. After treatment, the explants were washed with 1 × PBS and immediately transferred into T-PER lysis buffer (Thermo Fisher Scientific) containing protease and phosphatase inhibitors. The proteins were separated by electrophoresis, blotted onto PVDF membrane, and probed first with primary antibodies and subsequently with corresponding peroxidase-conjugated secondary antibodies. The bands were visualized by chemiluminescence using West Femto Super Signal (Thermo Fisher Scientific). Immunoblots were always analyzed for the phosphorylated form of proteins first, then stripped with Restore PLUS Western Blot Stripping Buffer (Thermo Fisher Scientific) and re-probed for the corresponding total protein. GAPDH was used as a loading control. We calculated the relative amounts of Akt and c-Jun proteins on the blots. The relative densities of specific proteins were calculated using ImageJ software (NIH) and normalized against untreated WT control.

### Statistical methods

We used two-way analysis of variance (ANOVA) followed by Bonferroni's multiple comparison test to analyze the difference between groups (GraphPad PRISM, GraphPad software, La Jolla, CA, USA). *P*-values <0.05 were considered statistically significant.

## Abbreviations

3-MA: 3-methyladenine
B: brain
GAPDH: glyceraldehyde-3-phosphate dehydrogenase
IL-6: interleukin 6
IL-10: interleukin 10
JAK2: Janus kinase-2
JNK: c-Jun N-terminal kinase
LC3: microtubule-associated protein 1 light chain 3
M: modiolus
NF-*κ*B: nuclear factor kappa B
OC: organ of Corti
PCR: polymerase chain reaction
PI3K: phosphatidylinositol 3-kinase
siRNA: short interfering RNA
SG: spiral ganglion
SV: stria vascularis
STAT1: signal transducer and activator of transcription-1
TUNEL: Terminal deoxynucleotidyl transferase dUTP nick end labeling
TNF-*α*: tumor necrosis factor-alpha
